# Supplementary material for: A Novel Indole Derivative with Superior Photophysical Performance for Fluorescent Probe, pH-Sensing, and Logic Gates
Source: Int J Mol Sci. 2023 Jan 15;24(2):1711. doi: 10.3390/ijms24021711 (PMC9860819; doi:10.3390/ijms24021711)
Supplement: Supplementary file 1 [file ijms-24-01711-s001.zip › ijms-1956891-supplementary.pdf]

A Novel Indole Derivative with Superior Photophysical Performance for Fluorescent Probe, pH-Sensing, and Logic Gates

Hai-Ling Liu[1,2], Kan Zhan[3,*], Kai-Liang Zhong[1,*] , Xing-Liang Chen[1], Xing-Hua Xia[2]

[1]School of Chemistry and Chemical Engineering, Shaoxing University, Shaoxing 312000, China

[2]State Key Laboratory of Analytical Chemistry for Life Science, School of Chemistry and Chemical Engineering; Nanjing University, Nanjing 210023, China

[3]College of Biotechnology and Bioengineering, Zhejiang University of Technology, Hangzhou 310014, China

Table of Contents

1. Crystallographic Data for **PI**.
2. Crystallographic Data for **NI**.
3. Crystallographic Data for **TI**.
4. Crystallographic Data for **FI**.
5. NMR Spectra

Table S1 Crystallographic Data for **PI**.

Formula sum	C_{19} H_{21} N_1 O_1
Space-group	Cc
Cell parameters	a=13.659 Å b=15.138Å c=8.158 Å α=90° β=111.075(2)° γ=90°

CCDC deposition number: 1842009

Table S2 Crystallographic Data for **NI**.

Formula sum	C_{19} H_{22} N_2
Space-group	Cc
Cell parameters	a=13.634Å b=15.386Å c=8.332 Å α=90° β=117.504(4)° γ=90°

CCDC deposition number: 1842008

Table S3 Crystallographic Data for **TI**.

Formula sum	C_{29} H_{26} N_2
Space-group	P21/c
Cell parameters	a=11.26Å b=14.997Å c=13.15 Å α=90° β=92.035(3)° γ=90°

CCDC deposition number: 1842010

Table S4 Crystallographic Data for **FI.**

Formula sum	$C_{18}H_{16}$ F_3 N_1
Space-group	C2/c
Cell parameters	a=30.843 Å b=6.528Å c=17.909 Å α=90° β=122.058(3)° γ=90°

CCDC deposition number: 1842007

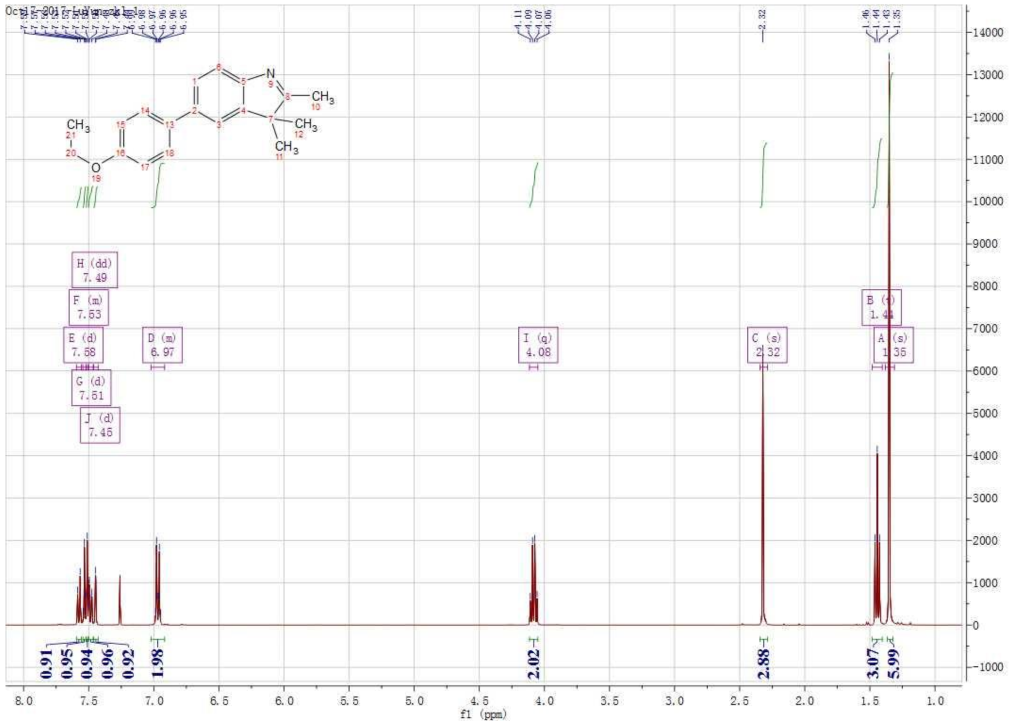

Figure S1. The ^{1}H NMR spectrum of **PI**.

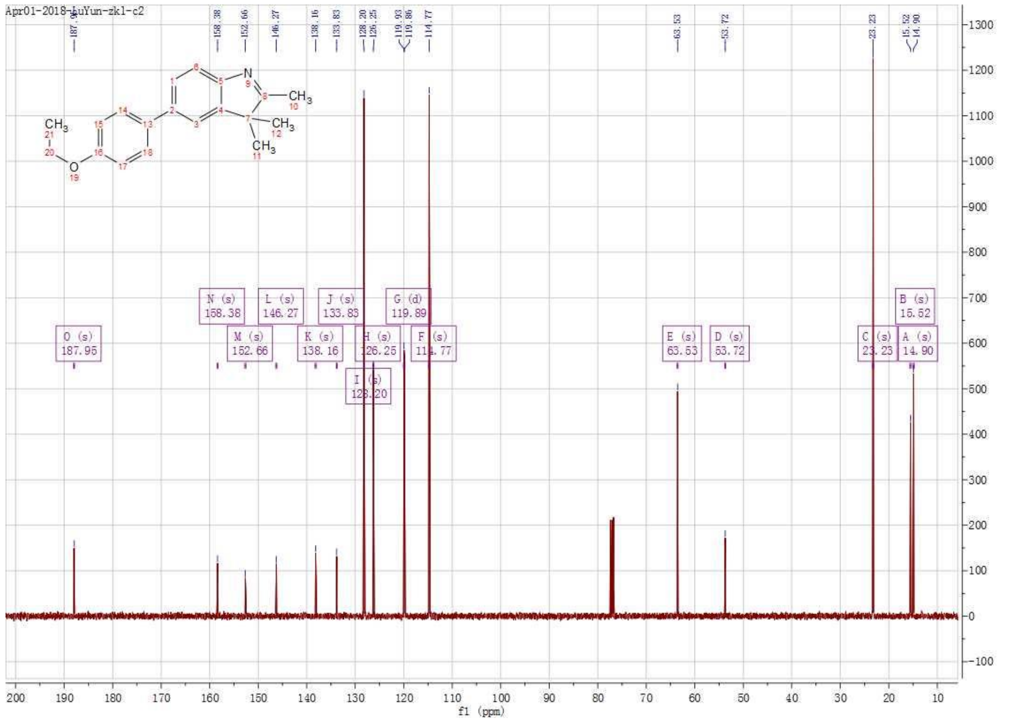

Figure S2. The ^{13}C NMR spectrum of **PI**.

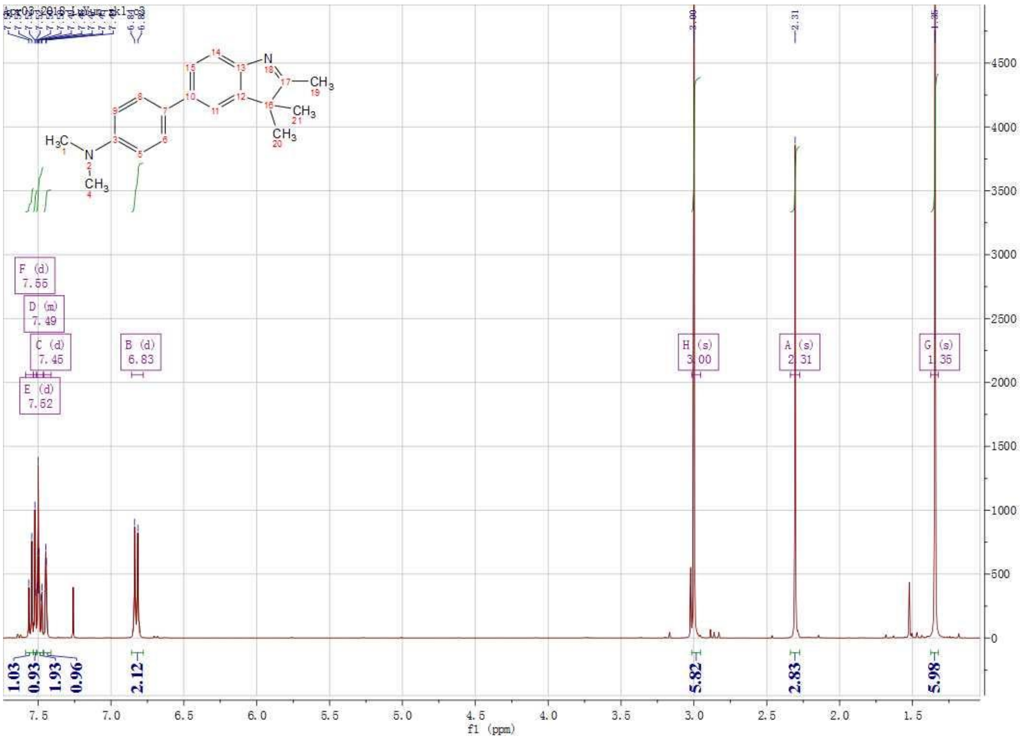

Figure S3 The ^{1}H NMR spectrum of **NI**.

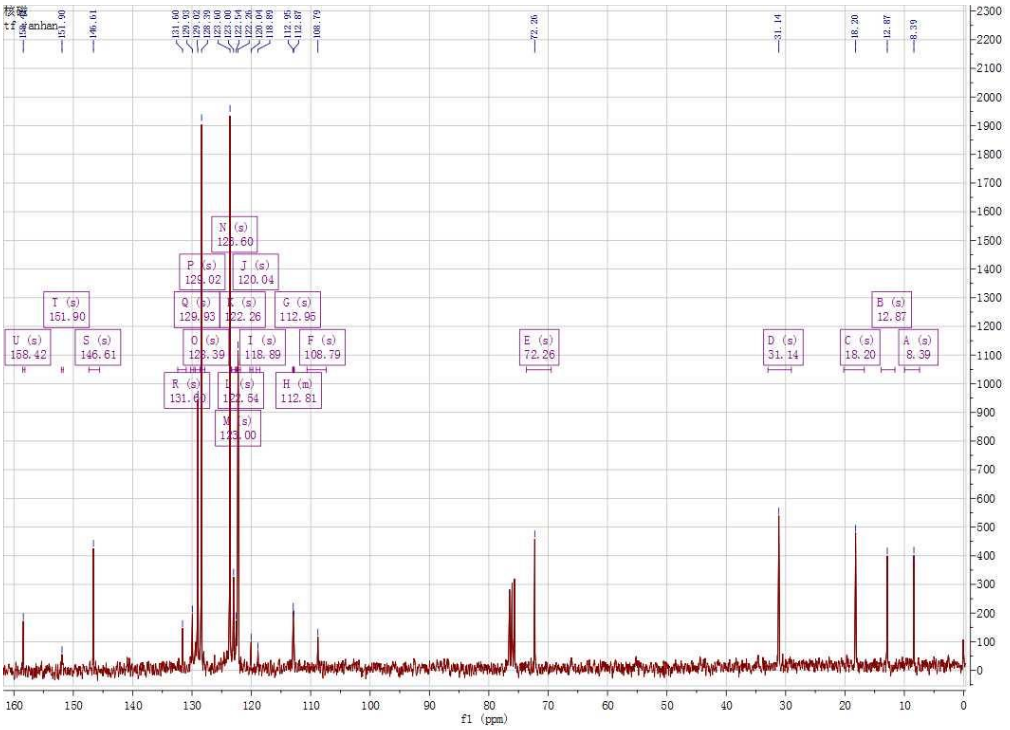

Figure S4. The ^{13}C NMR spectrum of **NI**.

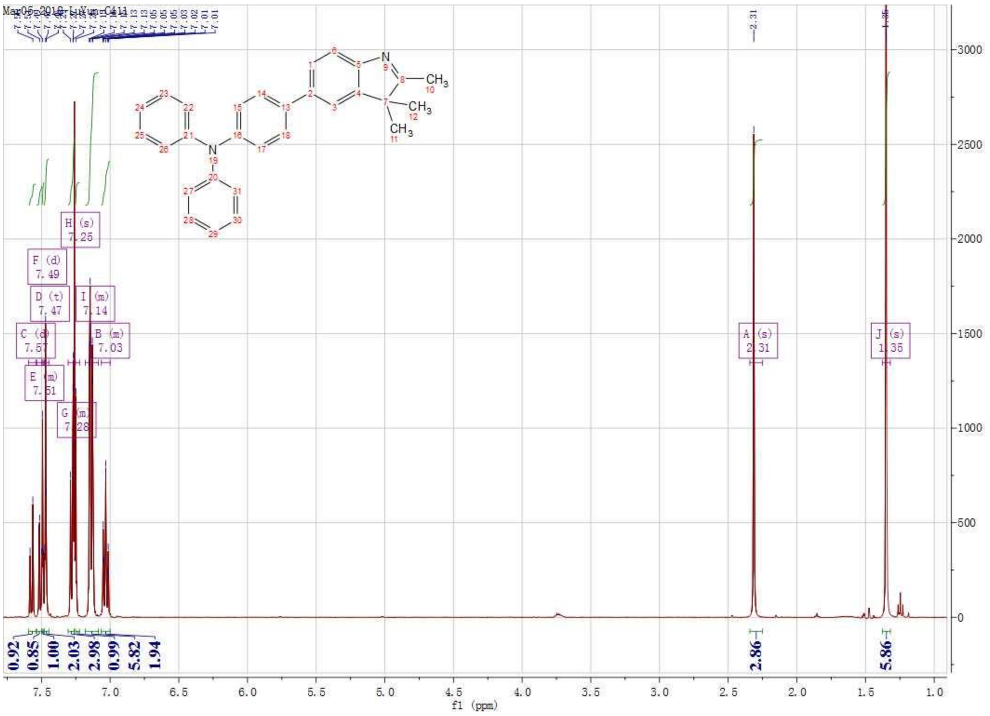

Figure S5. The ^{1}H NMR spectrum of **TI**.

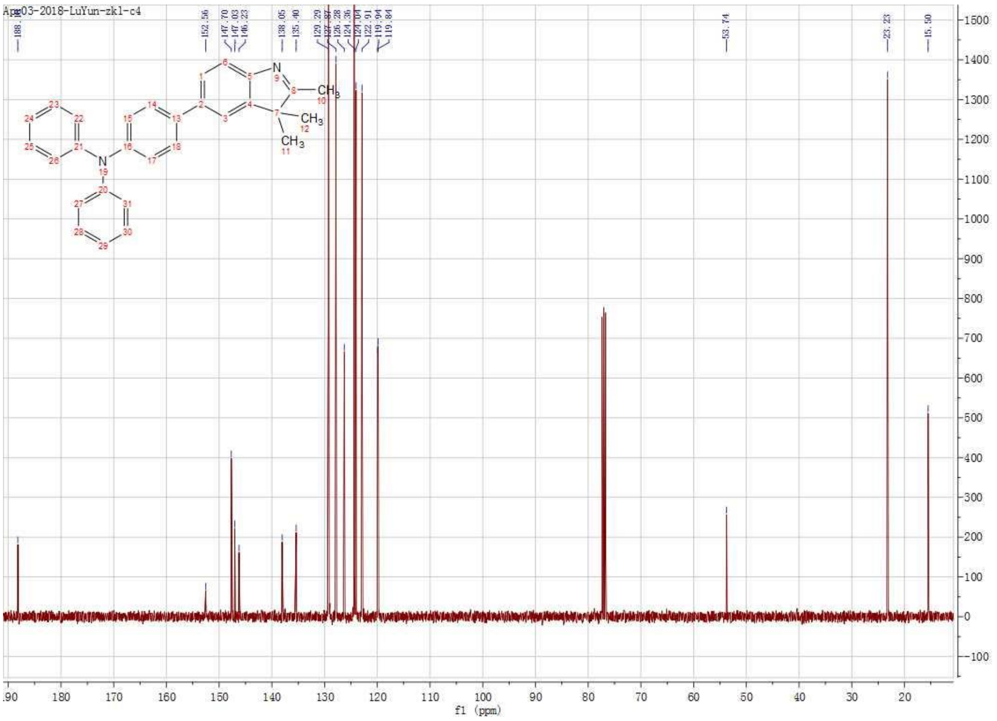

Figure S6. The ^{13}C NMR spectrum of **TI**.

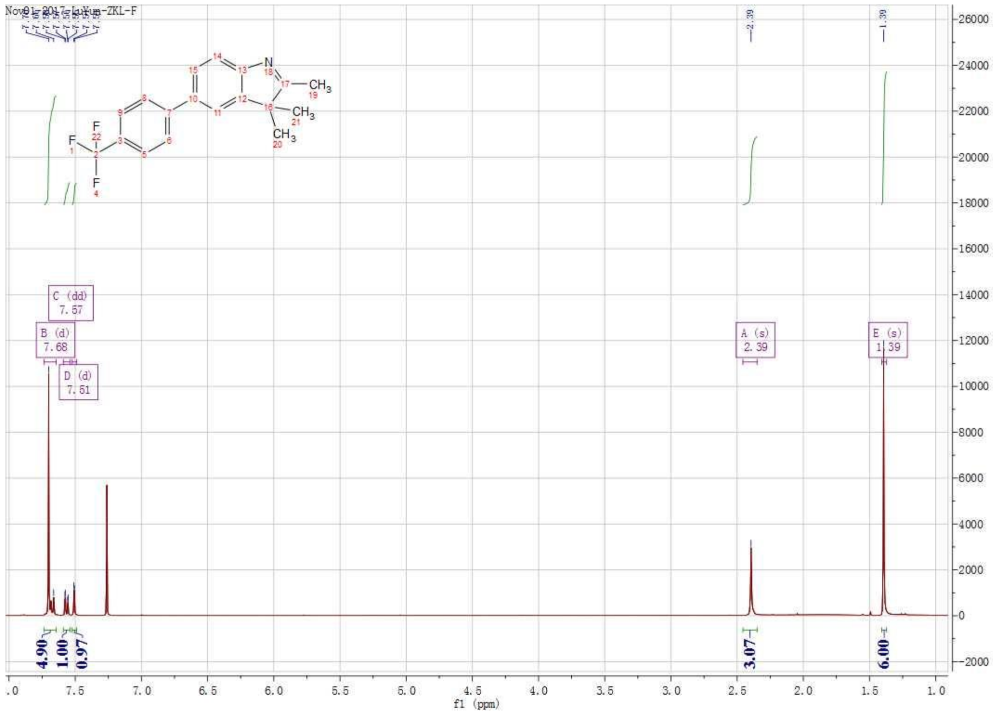

Figure S7. The ^{1}H NMR spectrum of **FI**.

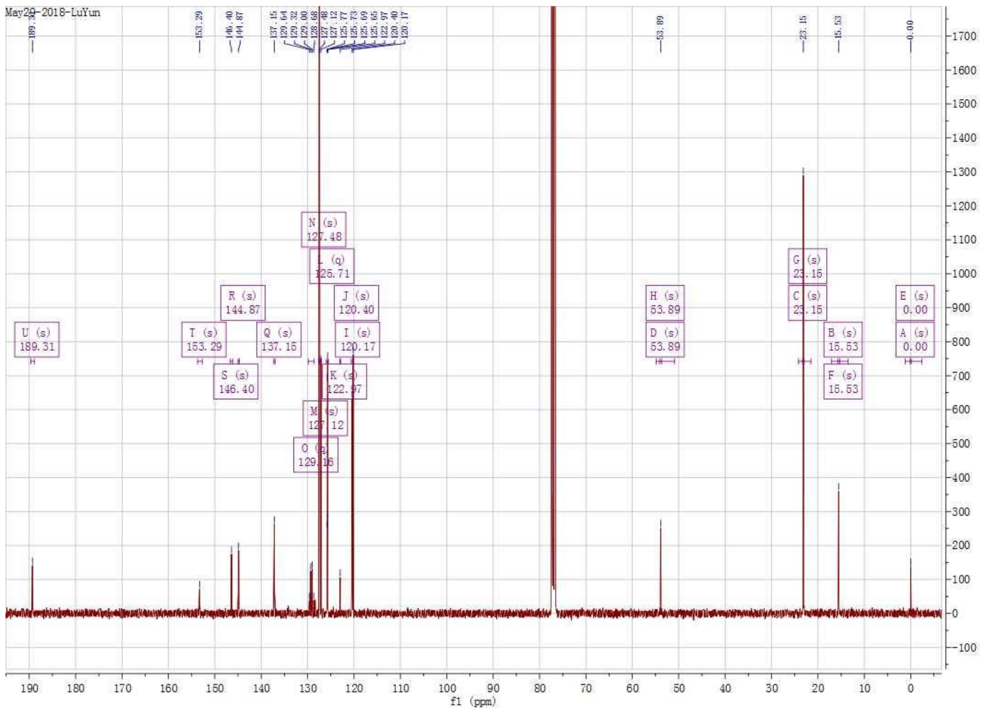

Figure S8. The ^{13}C NMR spectrum of **FI**.
